# Mushroom lectin overcomes hepatitis B virus tolerance via TLR6 signaling

**DOI:** 10.1038/s41598-017-06261-5

**Published:** 2017-07-19

**Authors:** Meina He, Dan Su, Qinghong Liu, Wenjuan Gao, Youmin Kang

**Affiliations:** State Key Laboratory for Agro-Biotechnology, College of Biological Science, China Agricultural University, Beijing, China

## Abstract

Currently, chronic hepatitis B virus (HBV) infection remains a serious public health problem in the world. Recombinant HBV vaccine, as a preventive strategy against HBV infection, generates high antibody level, but it is not effective to activate innate and cellular immunity for chronic HBV infection therapy. Lectins from mushroom are natural and active proteins which have been shown important biological functions. However, little is known about the immunological mechanism engaged by mushroom lectins. Here we report that, lectin from *Pleurotus ostreatus* (POL) stimulated innate response by activating Toll-like receptor 6 signal pathway of dendritic cells. Subsequently POL enhanced HBV specific antibody level and follicular helper T cells response which overcame HBV tolerance in transgenic mice. This study suggests a novel mechanism for POL acting on immune response and a therapeutic approach to break HBV tolerance.

## Introduction

Chronic hepatitis B virus (HBV) infection is a serious disease that causes public health problems worldwide^1, 2^. Accumulated data have shown that a recombinant HBV vaccine with an alum adjuvant generates high antibody level and is a promising strategy for activating immune response and protecting against HBV infection in humans^2, 3^. Alum (Al) is the most widely used adjuvant in humans because it primarily elicits a T helper 2 (Th2) cell mediated response and has a good safety record. For almost one century, alum has been the only adjuvant approved and licensed for human vaccine by the U.S. Food and Drug Administration^4, 5^. However, HBV vaccine containing alum as an adjuvant and recombinant HBV surface antigen (HBVsAg) are not effective for chronic HBV infection since it does not elicit an effective cellular immune response and has no therapeutic effect in chronic HBV carriers^6^. Currently available therapies fail to control viral replication in most patients^2, 7^.

Several methods, including adjuvants, have been suggested to enhance the immune response generated by recombinant HBV vaccine^8^. Levamisole is an antihelminthic drug that stimulates T-cell response^9^. In one study, dialysis patients showed a significant improvement in immune response to HBV vaccine when levamisole was used as an adjuvant; however, the limited number of patients in the study limits the conclusions that could be drawn^10^. A combination of levamisole and an alum adjuvant has been shown to synergistically enhance the immunogenicity of HBVsAg^11^. In another study, HBV vaccine with granulocyte-macrophage colony-stimulating factor (GM-CSF) as an adjuvant elicited increased patient response rates compared with HBV vaccine alone^12, 13^. Administration of GM-CSF prior to vaccination with recombinant HBV vaccine produced high IgG level and stimulated CD8 T cellular response in HBV-transgenic mice^14^. A formulation comprising recombinant HBV and a CpG oligonucleotide (1018 ISS) has been shown to induce a robust humoral and cell mediated immunity against HBV^15^. Heat shock protein gp96 enhanced immune responses and potentiates the anti-HBV activity in BALB/c and transgenic mice^16^.

Lectins induce cell agglutination and have been shown to be possessed in important biological processes^17, 18^. Lectins are abundant in mushrooms, and a variety of lectins have been isolated from edible mushrooms^19–22^. Although several mushroom lectins have been purified and characterized, only some have been shown to possess immunological activity^23, 24^. Some mushroom lectins showed mitogenic activities towards mouse T cells^25^. Lectin from *Pleurotus ostreatus* (POL) has high antitumor activity^26^. Our previous study showed that POL as an adjuvant in an HBV DNA vaccine activated strong Th2 and cytotoxic T cell 1 (Tc1) responses^27^.

Innate immunity plays a major role in host defense during the early stages of infection. The first step in innate immunity is the recognition of microbes by receptors including toll-like receptors (TLRs)^28^. C-type lectins are a type of pattern recognition receptor, which mostly recognize carbohydrate structures in pathogens. TLRs are a family of ten microbe-recognition receptors that are important to mediate effective innate immune response^29^. TLRs generate intracellular signals with the potential to elicit inflammatory responses. Little is known about the effect of mushroom lectins on innate immunity. In this study, we report for the first time the activation of innate immunity by POL for treatment of chronic HBV infection.

## Results

### POL increased HBV-specific cellular immune response in immunized C57BL/6 mice

**Figure 1:**
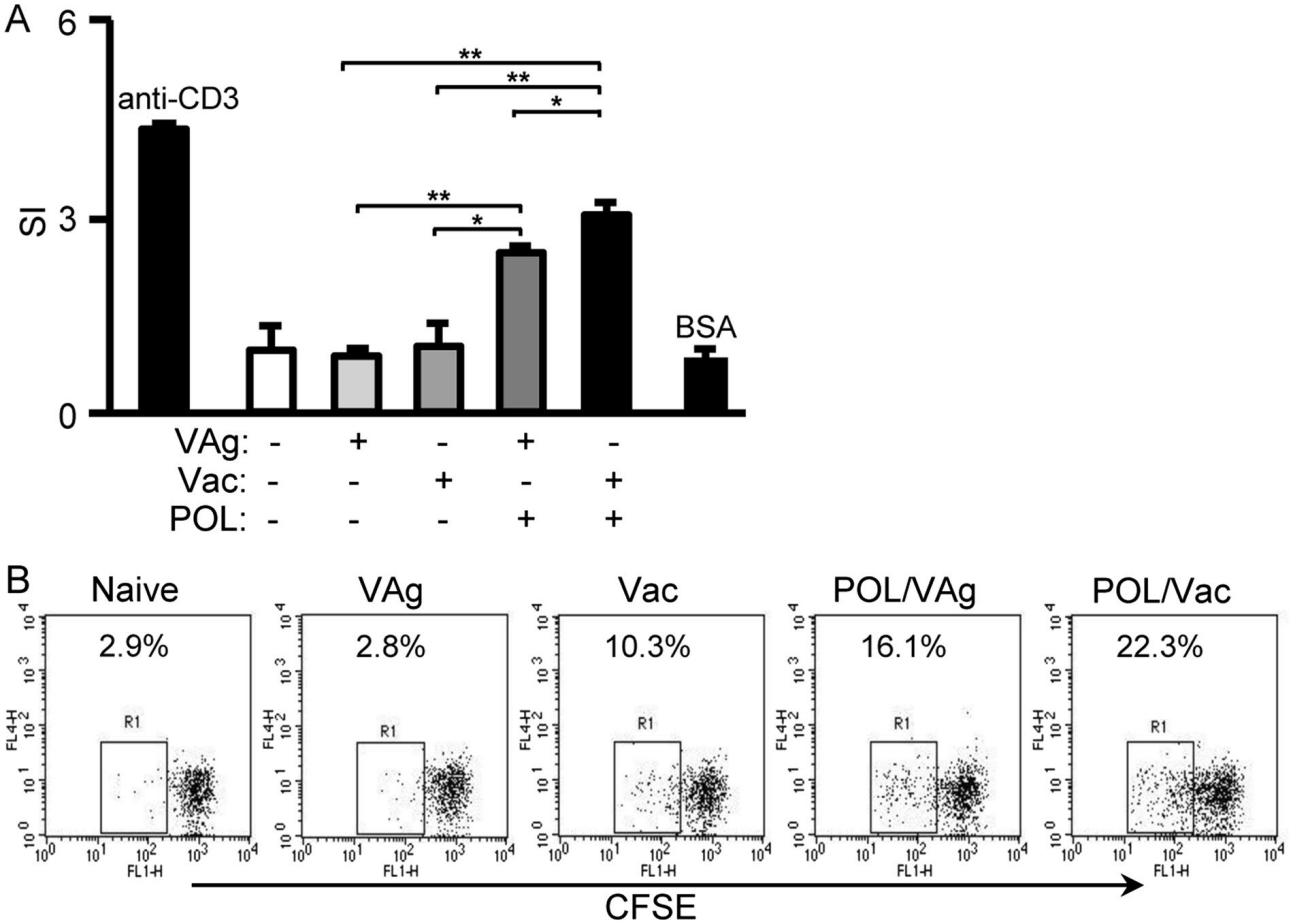
POL stimulated HBV specific T cell response in C57BL/6 mice. (**A**) 4 days after the 3^rd^ vaccination, the splenocytes of immunized mice were prepared. T cell proliferation was analyzed by the MTT method and expressed as SI. The anti-CD3 mAb was used as positive control while BSA was used as non-specific control. (**B**) 4 days after the 3^rd^ vaccination, the splenocytes of immunized mice were prepared. T cell proliferation was analyzed by CFSE staining method and the proliferative cells were analyzed. The anti-CD3 mAb was used as positive control. Shown in each panel is 1 of at least 3 experiments with similar results. Bar, mean and SD from 3 independent experiments, each using at least three mice per group (n = 3); *p < 0.05; **p < 0.01.

C57BL/6 were randomly divided into five groups (n = 9 per group). Mice were injected intramuscularly with 2 µg recombinant HBVsAg vaccine antigen (VAg group), 2 µg recombinant HBV vaccine (Vac group), 2 µg recombinant HBVsAg vaccine antigen and 1 µg POL (POL/VAg group), 2 µg recombinant HBVsAg vaccine and 1 µg POL (POL/Vac group). A control group was injected with saline. The mice were immunized on day 0 and boosted on days 14 and 28. All experiments were repeated three times. The injection sites exhibited no erythema or edema, and all mice appeared healthy after the injections. To check the cellular response stimulated by POL, splenocytes of immunized mice were prepared for T-cell proliferation analysis by MTT (demonstrated by 3-(4,5-dimethylthiazol-2-yl)-2,5- diphenyltetrazolium bromide) (Fig. 1A) and CFSE (demonstrated by 5,6- carboxyfluorescein diacetate, succinimidyl ester) staining (Fig. 1B). The a-CD3 monoclonal antibody was used as a positive control and showed high stimulation index (SI) while bovine serum albumin (BSA) was used as a negative control and showed low stimulation index (SI). The SI of the POL/VAg group was significantly higher than that of the VAg (*p* < 0.01) and Vac (*p* < 0.05) groups. Furthermore, the SI of the POL/Vac group was higher than that of the VAg (*p* < 0.01), Vac (*p* < 0.01) and POL/VAg (*p* < 0.05) groups (Fig. 1A). Similarly, the percentage of proliferative cells (R1) in the POL/Vac group was higher than those in the other groups (Fig. 1B).

**Figure 2:**
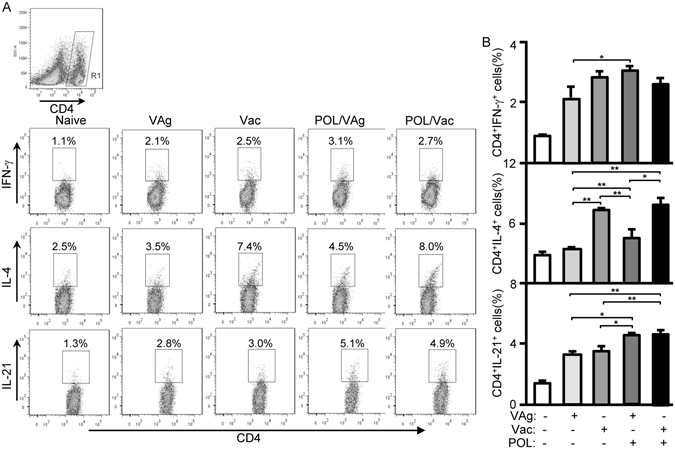
Cytokine levels of CD4 T cells in immunized C57BL/6 mice. 4 days after the 3^rd^ vaccination, the splenocytes of immunized C57BL/6 mice were prepared. For cytokines level in CD4 T cells analysis, the splenocytes were stimulated with Cell Stimulation Cocktail (eBioscience) and treated with Protein Transport Inhibitor Cocktail (eBioscience) for cytokines production by flow cytometry. (**A**) The samples were intracellularly stained with anti-mouse CD4-FITC/IFN-γ-PE for Th1 cytokine analysis, anti-mouse CD4-FITC/IL-4-PE for Th2 cytokine analysis or anti-mouse CD4-FITC/IL-21-PE for Tfh cytokine analysis. The CD4^+^IFN-γ^+^ cells, CD4^+^IL-4^+^cells or CD4^+^IL-21^+^ cells were counted relatively to total CD4^+^ T cells. (**B**) The statistical results of Th1, Th2 and Tfh cytokines were shown. Shown in each panel is 1 of at least 3 experiments with similar results. Bar, mean and SD from 3 independent experiments, each using at least three mice per group (n = 3); *p < 0.05; **p < 0.01.

Cytokines play important roles in the stimulation of immune response. To test the cytokine profiles of CD4 T-cells, splenocytes of immunized C57BL/6 mice were prepared and intracellularly stained for flow cytometry analysis. The CD4^+^ T cells were gated and CD4^+^IFN-γ^+^ CD4^+^ IL-4^+^ or CD4^+^IL-21^+^ cells were counted relatively to total CD4^+^ T cells (Fig. 2A). The POL/VAg group exhibited significantly elevated levels of Th1 cytokines (CD4^+^IFN-γ^+^) compared with the VAg group (*p* < 0.05), but showed no significant difference compared with the Vac or POL/Vac groups (Fig. 2B). Analysis of Th2 cytokine levels (CD4^+^ IL-4^+^) revealed that the Vac group produced significantly higher levels than the VAg (*p* < 0.01) and POL/VAg (*p* < 0.01) groups; the POL/VAg group produced significantly higher levels than the VAg group (*p* < 0.01); and the POL/Vac group produced significantly higher levels than the VAg (*p* < 0.01) and POL/VAg (*p* < 0.05) groups; but there was no significant difference between the POL/Vac and Vac groups (Fig. 2B). Analysis of IL-21 in CD4 T-cells revealed that the POL/VAg group produced significantly higher levels than the VAg (*p* < 0.05) and Vac (*p* < 0.05) groups; and the POL/Vac group produced significantly higher levels than the VAg (*p* < *0*.*01*) and Vac (*p* < 0.01) groups, but there was no significant difference between the POL/Vac and Vac groups (Fig. 2B).

### POL elicited a follicular helper T cell (Tfh) response in immunized C57BL/6 mice

**Figure 3:**
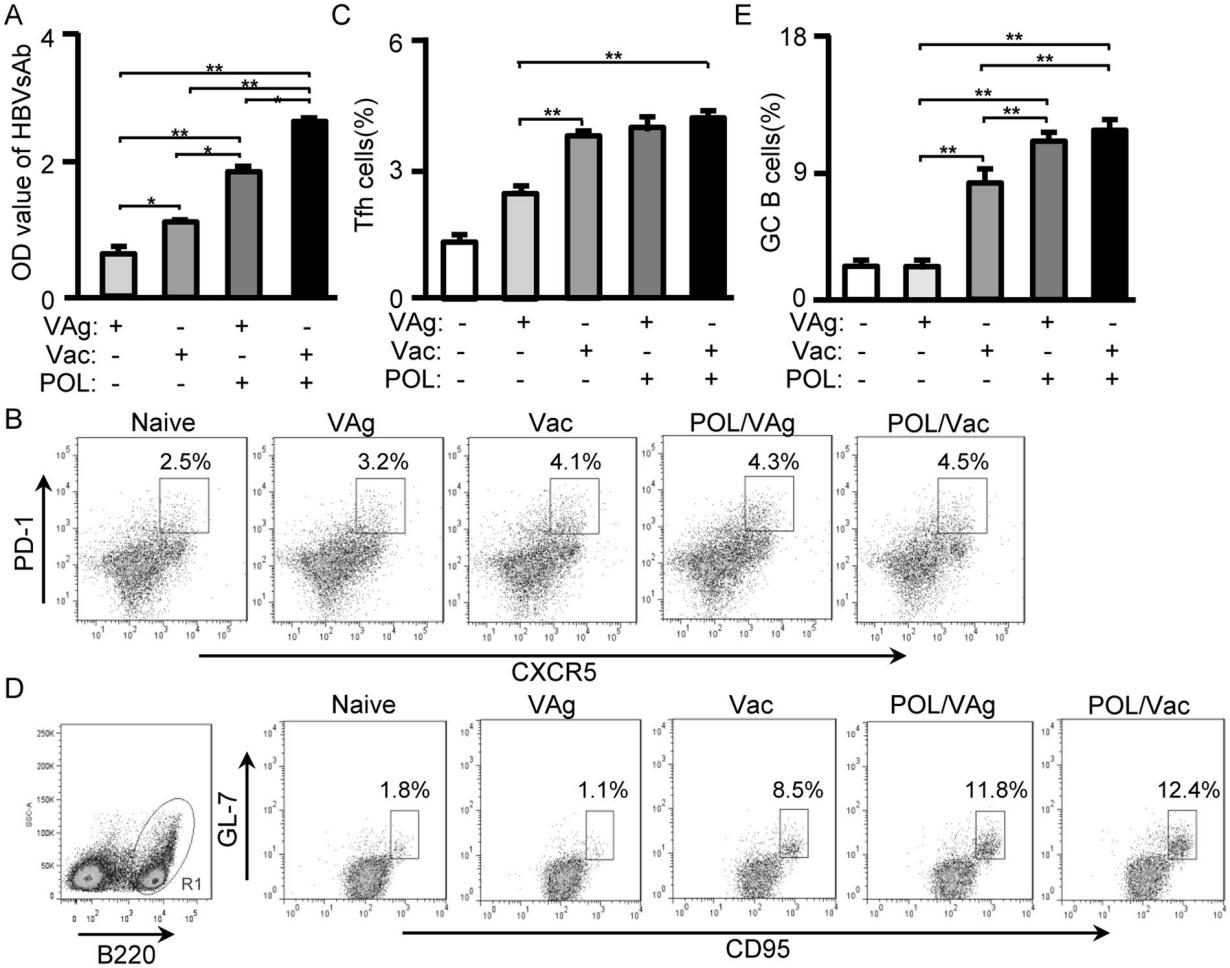
POL enhanced Tfh cell responses in immunized C57BL/6 mice. (**A**) 7 days after the 3^rd^ vaccination, the serum of immunized C57BL/6 mice were collected for HBV surface antibody level by using HBV surface antibody kit. (**B**) 7 days after the 3^rd^ vaccination, the splenocytes were stained with anti-mouse CD4-APC-Cy7/CXCR5-V450/PD-1-PE mAbs. The CD4^+^ cells were gated for Tfh cells analysis by flow cytometry. The CD4^+^CXCR5^+^ PD-1^+^ cells were counted relatively to total CD4^+^ cells. (**C**) The statistical results of Tfh cells were shown. (**D**) 7 days after the 3^rd^ vaccination, the splenocytes were stained with B220-PE-Cy5/CD95-APC/GL-7-FITC. The B220^+^ cells were gated for GC B cells analysis by flow cytometry. The B220^+^CD95^+^GL-7^+^ cells were counted relatively to total B220^+^ cells. (**E**) The statistical results of GC B cells were shown. Shown in each panel is 1 of at least 3 experiments with similar results. Bar, mean and SD from 3 independent experiments, each using at least three mice per group (n = 3); *p < 0.05; **p < 0.01.

As a Tfh cell-secreted cytokine, IL-21 is one of the most important B-cell stimulators and has functional roles in humoral immunity^30^. Because POL increased the level of IL-21 expression in CD4 T-cells (Fig. 2), IgG levels, Tfh cells and germinal center (GC) B-cells of immunized C57BL/6 mice were analyzed. Analysis of HBVsAb levels revealed that the Vac group exhibited significantly higher levels than the VAg group (*p* < 0.05); the POL/VAg group exhibited significantly higher levels than the VAg (*p* < *0*.*01*) and Vac (*p* < 0.01) groups; and the HBVsAb level in the POL/Vac group was the highest among all the groups (Fig. 3A). For Tfh cell evaluation, CD4^+^ T-cells were gated and Tfh cells (CD4^+^CXCR5^+^PD-1^+^) were analyzed. The percentage of Tfh cells was significantly higher in the Vac group than in the VAg group (*p* < 0.01); significantly higher in the POL/VAg group than in the VAg group (*p* < 0.01); and significantly higher in the POL/Vac group than in the VAg group (*p* < 0.01); but there were no significant differences in Tfh cell percentage among the Vac, POL/VAg and POL/Vac groups (Fig. 3B,C). For GC B-cell evaluation, B220^+^ cells were gated and GC B-cells (B220^+^CD95^+^GL-7^+^) were analyzed. The percentage of GC B-cells was significantly higher in the Vac group than in the VAg group (*p* < 0.01); significantly higher in the POL/VAg group than in the VAg and Vac groups (*p* < 0.01); and significantly higher in the POL/Vac group than in the VAg and Vac groups (*p* < 0.01); but there was no significant difference in GC B-cell percentage between the POL/VAg and POL/Vac groups (Fig. 3D,E).

### POL stimulated the TLR6 signaling pathway in dendritic cells (DCs) of immunized C57BL/6 mice

**Figure 4:**
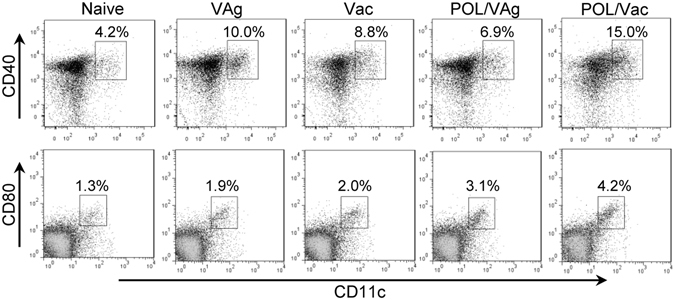
POL stimulated the maturation of DCs in immunized C57BL/6 mice. 3 days after the 3^rd^ vaccination, the splenocytes of immunized mice were prepared and stained for DCs analysis by flow cytometry. The samples were stained with anti-mouse CD11c-FITC/CD40-PE mAbs or CD11c-FITC/CD80-PE mAbs for DCs maturation. The CD11c^+^CD40^+^ cells or CD11c^+^CD80^+^ cells were counted relatively to total CD11c^+^ cells.

The major histocompatibility complex (MHC) molecules and co-stimulators play essential roles in DC maturation and innate immunity. To investigate DC maturation, splenocytes of immunized mice were stained with anti-mouse CD11c, CD40 and CD80 mAbs. CD40 and CD80 expression n DCs were increased in the POL/Vac group compared with other groups (Fig. 4), but there were no differences in MHCII or CD86 expression in DCs among all the groups (data not shown).

The first step in innate immunity is the recognition of microbes by different receptors. C-type lectins are pattern recognition receptors that mostly recognize carbohydrate structures in pathogens. C-type lectins, including mannose receptor (CD206), DCs-specific intercellular adhesion molecule-3-grabbing non-integrin, CD209 (DC-SIGN) and DCs associated C-type lectin 1 (Dectin-1, a specific receptor for β-glucans), activate the host innate immune system^31^. To assess whether POL can activate C-type lectins, splenocytes of immunized mice were stained with anti-mouse CD11c, CD206, CD209 and dectin-1 antibodies. There were no differences in CD206, CD209 or dectin-1 expression in DCs among any of the groups (data not shown).

**Figure 5:**
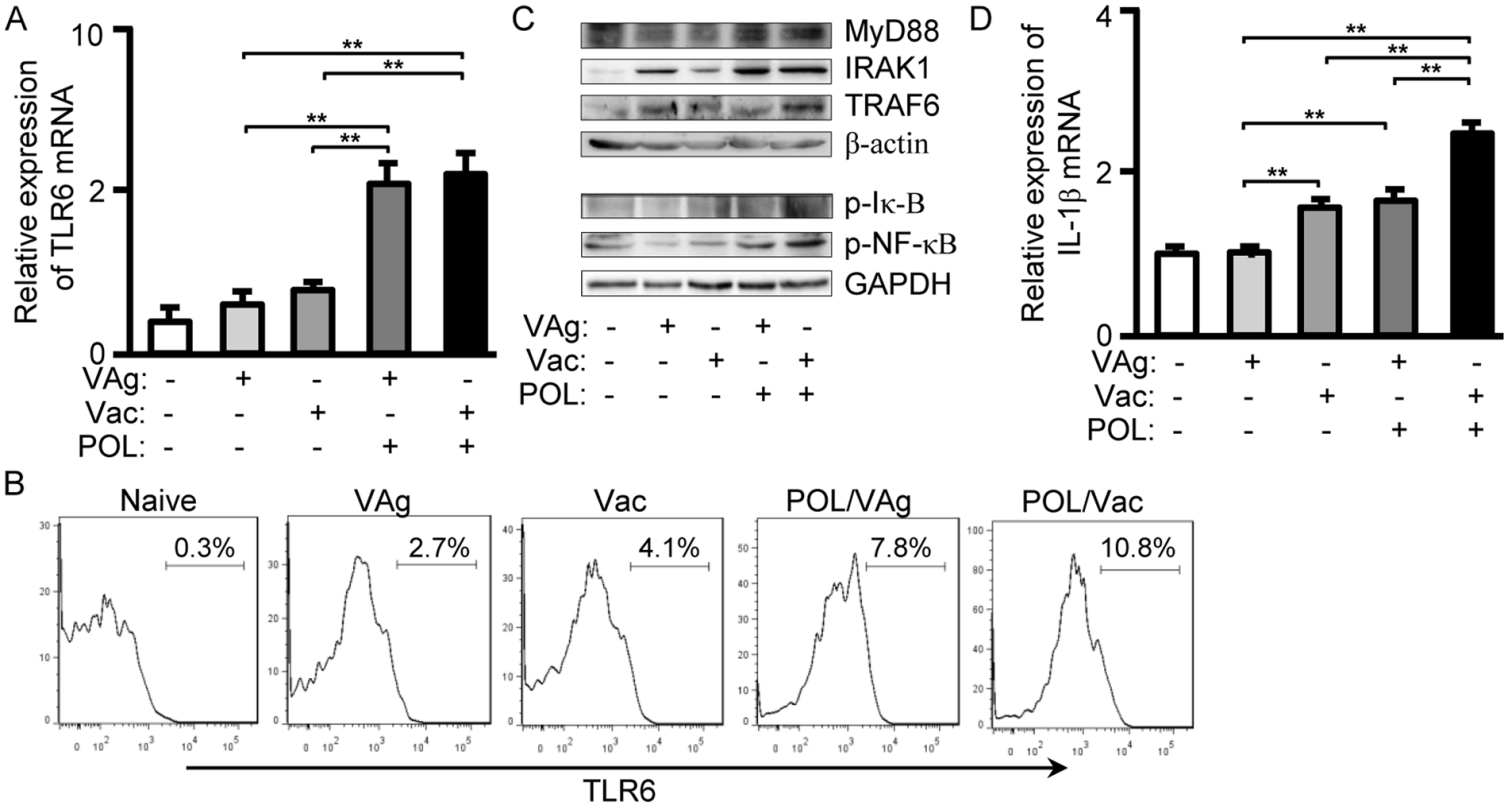
POL elicited TLR6 signaling pathway in immunized C57BL/6 mice. (**A**) 3 days after the 3^rd^ vaccination, total RNA from spleens was extracted and qPCR was performed for mRNA expression of TLR6. (**B**) 3 days after the 3^rd^ vaccination, the splenocytes were prepared and stained with anti-mouse CD11c-FITC/TLR6-APC mAbs. The CD11c^+^ cells were gated and CD11c^+^TLR6^+^ cells were counted relatively to total CD11c^+^ cells. (**C**) 3 days after the 3^rd^ vaccination, the splenocytes were prepared. The cytoplasm proteins or nuclei protein fractions were separated for western blot to examine the levels of MyD88, IRAK1, TRAF6, p-IκB and p-NF-κB. (**D**) 3 days after the 3^rd^ vaccination, total RNA from spleens was extracted and qPCR was performed for mRNA expression of IL-1β.

TLRs are a family of ten microbe-recognition receptors that are important to effective innate response^29^. TLRs generate intracellular signals that have the potential to elicit inflammatory responses. To assess whether TLRs can be activated by POL, quantitative PCR to evaluate TLR levels (TLRs 1–9, 11 and 12) were performed. TLR6 mRNA expression in the POL/VAg and POL/Vac groups was significantly upregulated, compared with in the VAg (*p* < 0.01) and Vac (*p* < 0.01) groups, but there was no significant difference between POL/VAg and POL/Vac groups (Fig. 5A). There were no differences of other TLRs expression among all the groups (data not shown). To confirm upregulation of TLR6, TLR6 protein levels were measured by flow cytometry. TLR6 expression was increased in the POL/VAg and POL/Vac groups (Fig. 5B). To examine the TLR6 signaling pathway, western blotting was performed to evaluate expression of the adaptor protein MyD88 (demonstrated by myeloid differentiation primary response gene 88) and the signal molecules IRAK1 (demonstrated by interleukin 1 receptor associated kinase 1), TRAF6 (demonstrated by TNF Receptor-Associated Factor 6), p-IκB (demonstrated by phosphorylated inhibitor of NF-κB) and p-NF-κB (demonstrated by phosphorylated Nuclear factor-kappa B). Expressions of these molecules were upregulated in the POL/VAg and POL/Vac groups compared with other groups (Fig. 5C). Similarly, IL-1β mRNA expression was significantly upregulated in the POL/Vac group compared with in the other groups (*p* < 0.01). IL-1β mRNA expression was significantly increased in the POL/VAg and Vac groups compared with the VAg group, but there was no significant difference between the POL/VAg and Vac groups (Fig. 5D).

### Therapeutic effect of POL in HBV-transgenic mice

**Figure 6:**
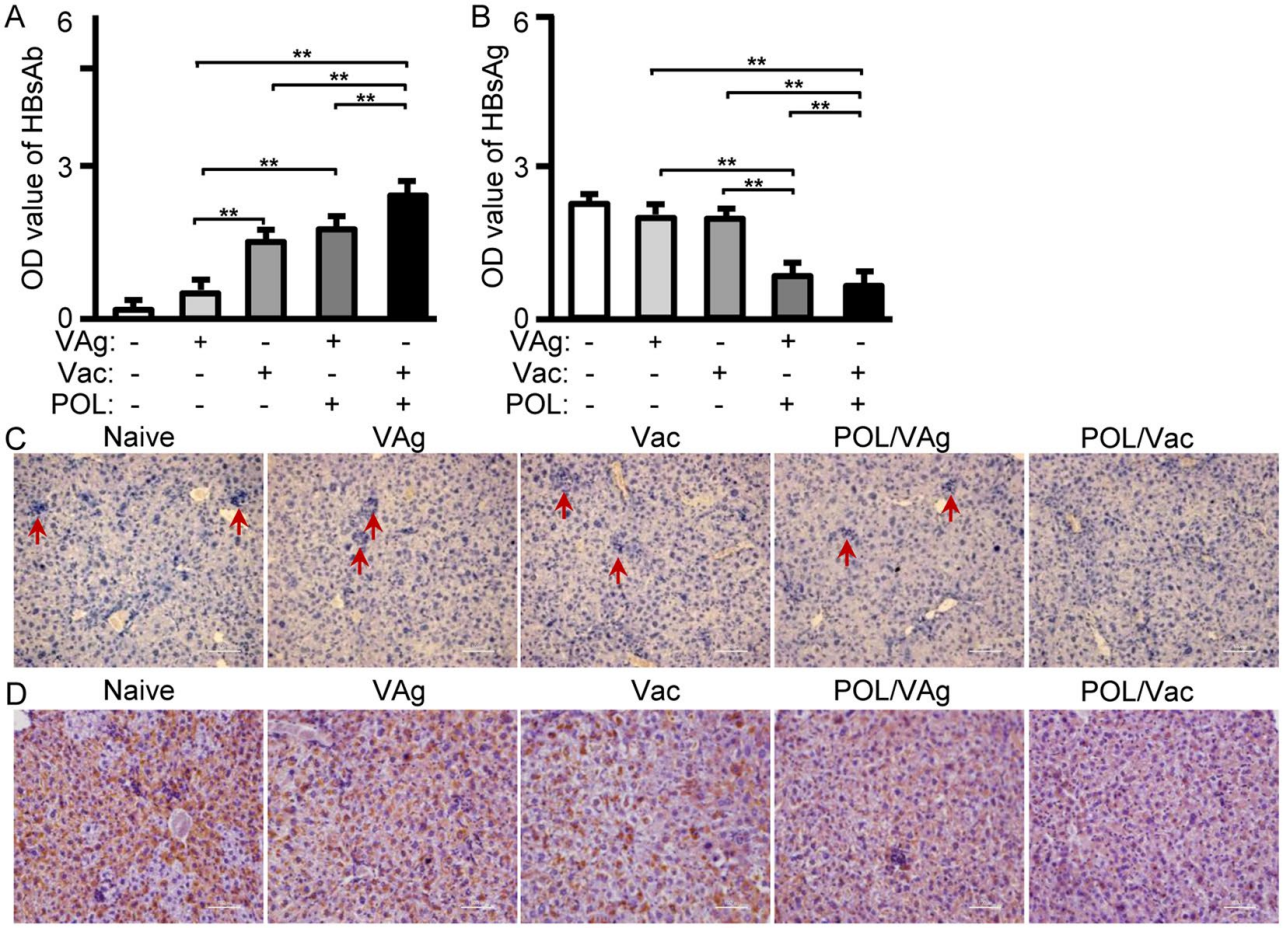
POL improved HBV specific liver pathological conditions in HBV -transgenic mice. (**A**) 7 days after the 3^rd^ treatment, the serum of HBV -transgenic mice were collected for HBV surface antibody level by using HBV surface antibody kit. (**B**) 7 days after the 3^rd^ treatment, the serum of HBV -transgenic mice were collected for HBV surface antigen level by using HBV surface antigen kit. (**C**) 7 days after the 3^rd^ treatment, the livers of treated mice were prepared for pathological sections by HE staining. (**D**) 7 days after the 3^rd^ treatment, the livers of treated mice were prepared for HBVsAg expression by immunohistochemistry staining. Shown in each panel is 1 of at least 3 experiments with similar results. Bar, mean and SD from 3 independent experiments, each using at least three mice per group (n = 3); *p < 0.05; **p < 0.01.

**Figure 7:**
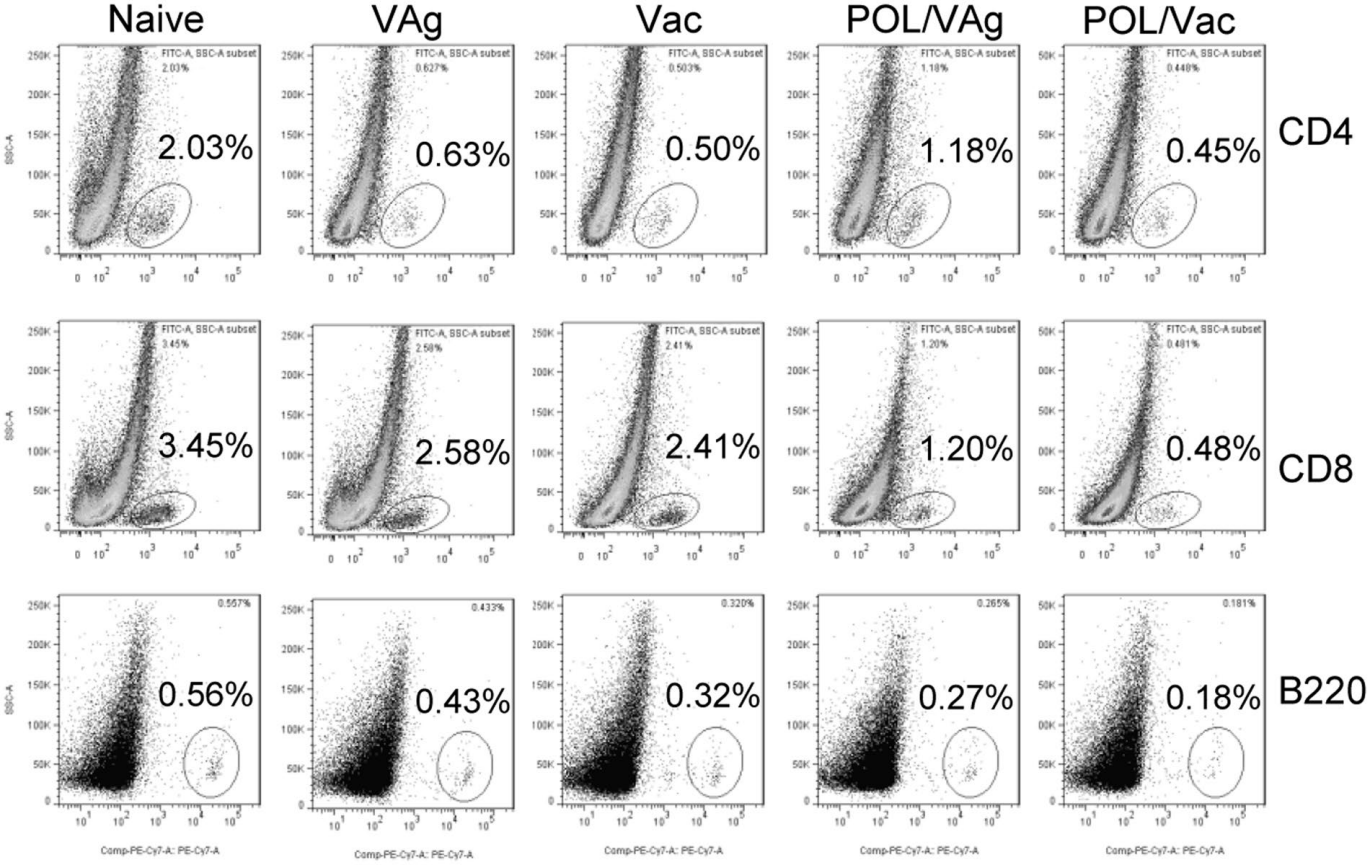
POL weakened lymphocyte infiltration in treated HBV -transgenic mice. 7 days after the 3^rd^ treatment, the splenocytes of treated mice were prepared stained with anti-mouse CD4-PE/CD8-PerCP/B220-PE-Cy7 mAbs for lymphocytes infiltration analysis by flow cytometry. The CD4^+^, CD8^+^ and B220^+^ cells were counted relatively to total liver cells. Shown in each panel is 1 of at least 3 experiments with similar results.

The HBVsAg-transgenic mouse constitutively produces large amounts of HBsAg in the liver, so it is considered a preclinical model for evaluating specific immunotherapy for HBV infection^7^. To confirm the immune effect of POL, HBVsAg-transgenic mice were treated with POL/VAg and POL/Vac. Untreated, and VAg- and Vac-treated HBVsAg-transgenic mice were used as controls. Seven days after the third treatment, sera were collected and HBsAg and HBVsAb levels were evaluated. Among the groups, the serum HBVsAb level was highest in the POL/Vac group (*p* < 0.01) (Fig. 6A). Serum HBVsAb levels were higher in the POL/VAg and Vac groups than in the VAg group (*p* < 0.01), but there were no differences between the POL/VAg and Vac groups. Among all of the groups, the serum HBVsAg level was lowest in the POL/Vac group (*p* < 0.01). The serum HBVsAg level in the POL/VAg group was lower than those in the VAg and Vac groups. There was no difference in serum HBVsAg level between the Vac and VAg groups (Fig. 6B). Similarly, the POL/Vac group exhibited the least lymphocyte infiltration (Fig. 6C) and the lowest HBVsAg expression in liver (Fig. 6D). The HBVsAg expression level in liver was lower in the POL/VAg group than in the Vac and VAg groups, consistent with serum HBVsAg levels (Fig. 6D). As shown in Fig. 7, the infiltrated lymphocytes of livers were analyzed by flow cytometry. The results showed that the infiltrated CD8^+^ T and B220^+^ B cells were decreased in POL/Vac group compared with other groups. There was no difference in CD4^+^ T cells infiltration between the Vac and VAg groups (Fig. 7).

### POL overcame tolerance by activating innate response in HBV-transgenic mice

**Figure 8:**
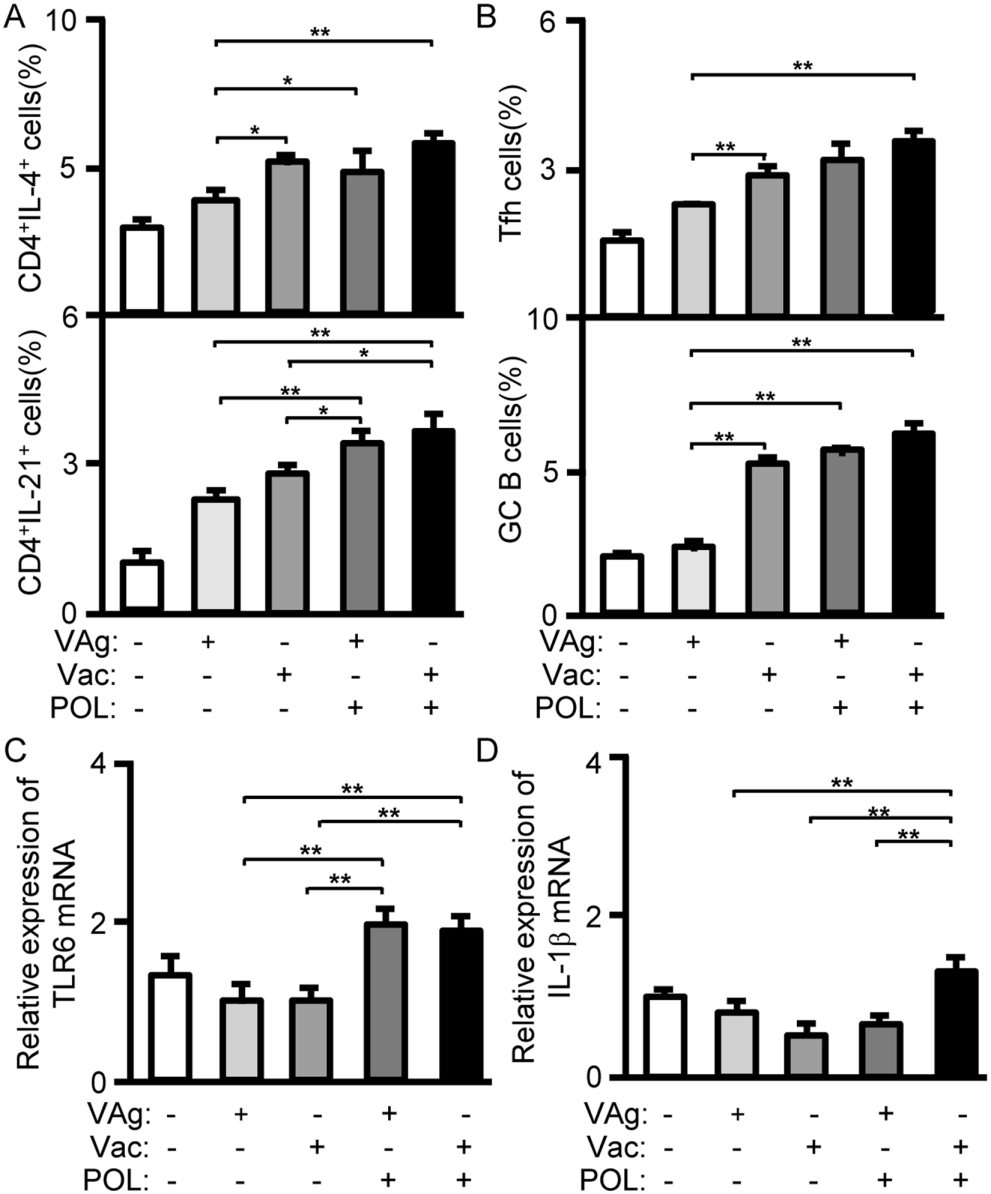
POL activated Tfh and innate responses in treated HBV -transgenic mice. (**A**) 7 days after the 3^rd^ treatment, the splenocytes of treated mice were prepared. The samples were stimulated with Cell Stimulation Cocktail (eBioscience, San Diego, CA) and treated with Protein Transport Inhibitor Cocktail (eBioscience, San Diego, CA). The samples were intracellularly stained with anti-mouse CD4-FITC/IL-4-PE and anti-mouse CD4-FITC/IL-21-PE for cytokine productions in CD4 T cells by flow cytometry. The CD4^+^IL-4^+^cells or CD4^+^IL-21^+^ cells were counted relatively to total CD4^+^ T cells. (**B**) 7 days after the 3^rd^ treatment, the splenocytes of treated mice were prepared. The samples were stained with anti-mouse CD4-APC-Cy7/CXCR5-V450/PD-1-PE mAbs for Tfh cells analysis and stained with B220-PE-Cy5/CD95-APC/GL-7-FITC for GC B cells analysis by flow cytometry. The Tfh cells and GC B cells were counted relatively to total CD4^+^ cells or total B220^+^ cells. (**C**) 3 days after the 3^rd^ treatment, total RNA from spleens was extracted and qPCR were performed for TLR6 expression. (**D**) 3 days after the 3^rd^ vaccination, total RNA from spleens was extracted and qPCR were performed for IL-1β expression. Shown in each panel is 1 of at least 3 experiments with similar results. Bar, mean and SD from 3 independent experiments, each using at least three mice per group (n = 3); *p < 0.05; **p < 0.01.

In HBV-transgenic mice, HBsAg as a self-antigen cannot stimulate specific immune response. To confirm the effect of POL on cellular immune response in HBV-transgenic mice, splenocytes of treated mice were intracellularly stained with anti-mouse CD4, IL-4 and IL-21 antibodies for cytokine analysis. Compared with in the VAg group, IL-4 expression was increased in the Vac (*p* < 0.05), POL/VAg (*p* < 0.05) and POL/Vac (*p* < 0.01) groups, but there were no significant differences among these three groups. The POL/Vac group exhibited the highest level of CD4 T-cell IL-21 expressions. The POL/VAg group produced more IL-21 than the VAg (*p* < 0.05) and Vac (*p* < 0.01) groups (Fig. 8A). The results for IL-4 and IL-21 in CD4 T-cells were consistent with those for serum.

Because POL affected Tfh and GC B-cells in C57BL/6 mice, the same experiments were performed in HBV-transgenic mice. In these mice, compared with the VAg group, the Vac (*p* < 0.01), POL/VAg (*p* < 0.01)or POL/Vac (*p* < 0.01) group exhibited higher levels of Tfh cell stimulation, but there were no differences among these three groups (Fig. 8B). The same results were observed for GC B-cells.

To confirm the effect of POL on innate immunity in HBV-transgenic mice, the TLR6 signaling pathway was analyzed. TLR6 mRNA expression levels were significantly higher in the POL/VAg and POL/Vac groups than in the VAg (*p* < 0.01) and Vac (*p* < 0.01) groups (Fig. 8C), consistent with the findings in C57BL/6 mice. IL-1β mRNA expression was upregulated in POL/Vac group compared with other groups (*p* < 0.01). There were no differences among the VAg, Vac and POL/VAg groups (Fig. 8D).

## Discussion

HBV is an infectious disease that causes chronic hepatitis and liver cirrhosis globally. A recombinant HBV vaccine with an alum adjuvant is widely used for protection against HBV infection^3^. However, this vaccine primarily induces a humoral response that is not effective for eradication of chronic HBV infection^32^. Many approaches have been developed to enhance the immune response to protein vaccines, particularly with respect to the choice of adjuvant^1^. We describe herein the use of a novel adjuvant combined with a recombinant HBV vaccine against chronic HBV infection. Our results show that POL, a lectin from *Pleurotus ostreatus*, activated the TLR6 signaling pathway and elicited HBV-specific Tfh responses for chronic HBV infection treatment.

Studies have shown that lectins play important roles in many biological activities^17^. Many mushroom lectins have been isolated and studied for their functions^23^. POL showed high antitumor activity against sarcoma and hepatoma in mouse models^26, 33^. Here, we found that POL combined with a recombinant HBV vaccine stimulated HBV-specific T-cell proliferation (Fig. 1). Tfh cells are important in humoral immunity, and IL-21 is important for Tfh cell generation^30, 34^. In this study, we found that POL stimulated IL-21 production, and activated Tfh cells and GC B-cells, which contributed to an increase in HBVsAg-specific IgG levels (Fig. 3). These results demonstrate the effect of POL on HBV-specific humoral immune responses.

Innate immunity is important in host defense during the early stages of HBV infection. As antigen presenting cells, DCs are essential for the initiation of innate immunity and activation of adaptive immunity^10^. In this study, we found that POL stimulated DCs maturation by increasing CD40 and CD80 expression (Fig. 4). DCs recognize different microbes via different receptors^29^. TLRs are important receptors and generate intracellular signals for effective innate response^35^. Little is known about the effect of mushroom lectin on innate immunity. Here, we report for the first time that POL activated the TLR6 signaling pathway by upregulating expression of TLR6 and its related signal molecules, MyD88, IRAK1, TRAF6, IκB, NF-κB, and also of the inflammatory factor IL-1β (Fig. 5).

The HBVsAg-transgenic mouse is considered a preclinical model of HBV infection because it constitutively expresses HBVsAg in the liver and mimics healthy human chronic HBVsAg carriers^7, 36^. Many approaches to treat chronic HBV infection have been studied. In one study, levamisole used as an adjuvant in an recombinant HBV vaccine improved specific immune response in dialysis patients; however, the small number of study participants limited the conclusions that could be drawn^12^. In another study, GM-CSF increased response rates among HBV-infected patients when it was combined with a recombinant HBV vaccine^12^. 1018 ISS elicited seroprotective antibodies with fewer vaccinations than recombinant HBV vaccine only in a Phase III clinical trial^5^. In another study, inclusion of alum in a recombinant HBV vaccine stimulated Th2 responses, but had no therapeutic effect on chronic HBV infection^4^. Clearing persistent extracellular antigen of HBV could be an immunomodulatory strategy to reverse tolerance for an HBV infection therapy^37^. In this study, HBVsAg-transgenic mice were treated with POL/Vac; this combination showed a promising therapeutic effect. HBV-specific IgG levels were increased and HBVsAg levels were decreased significantly in treated HBVsAg-transgenic mice. Similarly, lymphocyte infiltration, particularly of CD8 T- and B-cells in the liver, was significantly decreased in treated HBVsAg-transgenic mice (Fig. 6). In POL-treated HBVsAg-transgenic mice, POL stimulated Tfh responses (Fig. 8B) consistent with those observed in immunized C57BL/6 mice (Fig. 6). The TLR6 signaling pathway was also activated in POL-treated HBVsAg-transgenic mice (Fig. 8C), consistent with findings in immunized C57BL/6 mice (Fig. 6). In summary, the results demonstrated that POL may interact with TLR6 and elicit HBV specific immune response. TLR6 expression and downstream signal molecules were checked by flow cytometry and qPCR methods in this study. The effect of POL on TLR6 signaling pathway will be confirmed by using TLR6 knockout mice and the specific binding of POL with TLR6 protein on the DC surface will be investigated in future studies.

In conclusion, our results show for the first time that POL can activate the TLR6 signaling pathway and stimulate Tfh responses for HBV-specific antibody production to treat chronic HBV infection.

## Materials and Methods

### Animals and reagents

Female C57BL/6 and HBV-transgenic mice (aged 6–8 weeks) were purchased from the Animal Institute of the Chinese Medical Academy (Beijing, China). All mice were maintained in a specific-pathogen-free facility under a 12 h/12 h light/dark cycle, and provided with pathogen-free food and water. All animal experimental protocols were performed in accordance with the Guide for the Care and Use of Laboratory Animals prepared by the Institutional Animal Care and Use Committee of China Agricultural University. All animal protocols (SKLAB-2016-01) were approved by the Animal Welfare Committee of China Agricultural University.

All antibodies for FACS analysis were purchased from eBioscience (San Diego, CA). POL was kindly provided by Dr. Qinghong Liu (China Agricultural University, Beijing, China). The recombinant HBV vaccine (Vac) and recombinant HBV vaccine antigen (VAg) were purchased from Beijing Tiantan Biological Products Co., Ltd (Beijing, China).

### Immunization and treatment

C57BL/6 and HBV-transgenic mice were randomly divided into five groups (n = 9 per group). Mice were injected intramuscularly with 2 µg HBVsAg (VAg group), 2 µg VAG in 100 µl Recombinant HBV vaccine (including alum adjuvant, Vac group), 2 µg VAg and1µg POL (POL/VAg group), or 1 µg POL combined with 100 µl recombinant HBV vaccine (POL/Vac group). A control group was injected with saline. The mice were immunized on day 0 and boosted on days 14 and 28. All experiments were repeated three times. The injection sites exhibited no erythema or edema, and all mice appeared healthy after the injections.

### T-cell proliferation analysis

Four days after the third immunization, three mice from each group were sacrificed and single lymphocyte suspensions were prepared from the spleen. T-cell proliferation analysis was performed using MTT staining (Sigma, St. Louis, MO) as described previously^9^ with VAg protein as a specific stimulator, anti-CD3 monoclonal antibody (mAb) as a positive control and BSA as an irrelevant antigen control. At the same time, the splenocytes were stained with CFSE (Invitrogen, Carlsbad, CA) and cultured with VAg protein for 3 days. The cells were then collected and cell division was analyzed by flow cytometry. CFSE-labeled cells were counted relative to total splenocyte numbers.

### Flow cytometry

For surface molecule staining, splenocytes were blocked with anti-CD16/32 mAb then immunostained with anti-mouse CD4, CD8 and B220 antibodies for lymphocyte analysis; with anti-mouse CD4, CXCR5 and PD-1 antibodies for follicular helper T cells (Tfh) cell analysis; with anti-mouse B220, GL-7 and CD95 antibodies for germinal center (GC) B-cell analysis; with anti-mouse CD11c, CD40 and CD80 mAbs for dendritic cells (DCs) analysis; and with anti-mouse CD11c, CD206, CD209, Dectin-1 and TLR6 antibodies for DCs receptor analysis.

For intracellular staining, splenocytes were stimulated with Cell Stimulation Cocktail (eBioscience, San Diego, CA) and treated with Protein Transport Inhibitor Cocktail (eBioscience, San Diego, CA) to stimulate cytokine production. After stimulation for 8 h, the samples were treated with Fixation/Permeabilization Diluent (eBioscience, San Diego, CA), then intracellularly immunostained with anti-mouse CD4, IFN-γ, IL-4 and IL-21 mAbs for cytokine expression analysis by flow cytometry.

### Quantitative PCR

Three days after the third vaccination, total RNA from spleens of C57BL/6 and HBV-transgenic mice was prepared using an RNeasy Mini Kit (Qiagen, Germany) according to the manufacturer’s instructions. Total RNA was reverse transcribed for cDNA synthesis using transcriptase (Promega, Madison,WI) and oligo(dT)18 primer (Yingweijieji trading company ltd., Shanghai, China). Quantitative PCR was performed using a LightCycler 480 System (Roche, Basel, Switzerland) and primers as follows: TLR6 forward, 5′ TGAGCCAAGACAGAAAACCCA 3′; TLR6 reverse, 5′ GGGACATGAGTAAGGTTCCTGTT 3′; IL-1β forward, 5′ TGTAATGAAAGACGGCACACC 3′; IL-1β reverse, 5′ TCTTCTTTGGGTATTGCTTGG 3′; and for internal reference, ribosomal protein L9 (RPL9), forward, 5′ CTGAAGGTCAAAGGGAATGTGTTC 3′; RPL9 reverse, 5′ TGGTCAGCCAGGAGCTTCTTG 3′. All primers were synthesized by Yingweijieji trading company ltd. (Shanghai, China). The results are presented as relative expression, normalized to RPL9. Analyses were conducted in triplicate.

### Western blot

Three days after the third vaccination, splenocytes from immunized C57BL/6 and HBV-transgenic mice were lysed and centrifuged. The supernatants were mixed with Laemmli loading buffer and separated by sodium dodecyl sulfate polyacrylamide gel electrophoresis. The separated proteins were transferred onto polyvinylidene fluoride membranes (Millipore Corporation, Billerica, USA), and blotted with primary antibodies. Subsequently, membranes were washed and incubated with horseradish peroxidase-conjugated secondary antibodies. Protein bands were visualized by enhanced chemiluminescence (Pierce, Appleton, WI). Antibodies against MyD88, IRAK1, TRAF6, phospho-IκB and phospho-NF-κB were purchased from Santa Cruz Biotechnology, Inc (Texas).

### Liver histology analysis

On day seven after the third treatment, livers of HBV-transgenic mice were prepared and fixed in 4% paraformaldehyde for one week, then embedded in paraffin. Serial 8-µm-thick sections were cut and affixed to slides. The slides were then deparaffinized and stained with hematoxylin and eosin for morphologic analysis. For immunohistochemistry staining, the slides were deparaffinized and stained with anti-HBVsAg antibody and diaminobenzidine detection system (Beijing Zhongshan Jinqiao Biological Technology Co., Ltd, Beijing, China). Sections were analyzed under a light microscope to detect histological changes.

### Statistical analysis

The Student’s t-test was used to compare two groups. Analysis of variance was used for multi-group analysis. Data are expressed as means ± standard deviation. p < 0.05 was considered statistically significant.
